# Lessons on catatonia with or without delirium on the consultation-liaison psychiatry service in a Lebanese tertiary care center: a retrospective chart review of 36 cases of catatonia

**DOI:** 10.1186/s12888-025-07646-x

**Published:** 2025-11-28

**Authors:** Alfred Chabbouh, Roua Abbas, Majida Al Kurdi, Lea Daou, Yara El Frenn, Rawan Koleilat, Maha Makki, Hani Tamim, Rita Khoury

**Affiliations:** 1 Department of Psychiatry, Faculty of Medicine, American University of Beirut, 11-0236 Riad El Solh, Beirut, 1107 2020 Lebanon; 2 Clinical Research Institute, American University of Beirut, Beirut, Lebanon; 3 Department of Internal Medicine Faculty of Medicine, American University of Beirut, Beirut, Lebanon; 4 College of Medicine, Alfaisal University, Riyadh, Saudi Arabia

**Keywords:** Catatonia, Delirium, Consultation-Liaison, Medical, Psychiatry, Evolutionary

## Abstract

**Background:**

Catatonia in the physically ill is an under-investigated topic and its diagnosis remains challenging, especially when it presents with comorbid delirium. In this study, we described the presentations and correlates of confirmed cases of catatonia stratified by the presence or absence of comorbid delirium.

**Methods:**

We examined the electronic health records of 36 patients with catatonia due to general medical condition using the Diagnostic and Statistical Manual of Mental Disorders, Fifth Edition (DSM-5) criteria seen on the consultation-liaison Psychiatry service in a Lebanese tertiary care center. We collected variables pertaining to demographic and diagnostic information, past medical history, Bush-Francis Scale, catatonia signs and symptoms, as well as diagnostic investigations when performed.

**Results:**

The mean age was 55.5 ± 19.1 years, and 52% were males. 27% of patients had a history of a non-psychiatric brain disease, and 36% had a history of psychiatric diagnoses. 22% had a history of thrombotic disorders. 50% of the participants had comorbid delirium with catatonia (DeliCat syndrome). Patients with DeliCat were significantly older (64.3 ± 14.8 vs. 46.6 ± 19.1) and had significantly higher rates of history of thrombotic disorders (38% vs. 5%) compared to catatonia-only patients. Other paraclinical investigations did not differ statistically between the groups.

**Conclusions:**

The study highlights the overlap between delirium and catatonia. Based on existing literature, we propose a model that conceptualizes catatonia and delirium as maladaptive sickness responses related to neuroinflammation and the defense cascade. This model has the potential to explain the shared pathophysiology of the two syndromes, offering a framework for future investigations.

## Introduction

Catatonia is a psychomotor syndrome characterized by an alteration of motor activity secondary to acute brain dysfunction. It can be classified as hypokinetic or hyperkinetic catatonia. Staring, mutism, and decreased responsiveness to stimuli are often seen in hypokinetic catatonia while impulsivity, restlessness, and excessive non-goal-directed activity characterize hyperkinetic or excited catatonia. Parakinetic manifestations, like echophenomena, paliphenomena, and posturing can happen in both forms [1, 2]. Moreover, autonomic instability can develop leading to a life-threatening form of catatonia, called malignant catatonia (MC) [1, 2]. In practice, catatonia can be seen as a spectrum [3], , and patients’ symptoms can fluctuate between the two forms over time. Mounting evidence also suggests that neuroleptic malignant syndrome, delirious mania, and periodic catatonia also belong to the catatonia spectrum [2, 4, 5]. In addition, *delirium tremens*, a serious complication of alcohol withdrawal, has been hypothesized as a possible form of excited catatonia [6].

Catatonia (6A4Z) is often associated with brain disorders like neurodevelopmental and neurodegenerative disorders, meningitis, encephalitis (especially anti-N-methyl-D-aspartate receptor encephalitis), mood disorders, and schizophrenia [1, 2]. Another frequent cause of catatonia is substance-withdrawal catatonia following rapid discontinuation of certain agents, namely alcohol, benzodiazepines, and clozapine. On the other hand, antipsychotic-induced catatonia has also been described [2, 7]. Catatonia is an underdiagnosed syndrome that occurs in around 1 in 33 medical inpatients, and 1 in 5 to 1 in 20 psychiatry inpatients. The influence of different diagnostic systems and instruments on the prevalence of catatonia and delirium is an important limitation to consider [2].

Delirium (6D70.Z) is an acute syndrome defined by impaired attention and arousal and is associated with global cognitive dysfunction (e.g., altered mood, psychosis). It is also divided into hyperactive or hypoactive subtypes based on psychomotor activity alterations. An extracranial process (e.g., sepsis, metabolic derangements) most often triggers delirium, although an intracranial process (e.g., stroke) can also be the culprit. Intoxication or withdrawal from substances including alcohol can be associated with delirium [8].

Unlike the International Classification of Diseases Eleventh Revision (ICD-11) [9], the Diagnostic and Statistical Manual of Mental Disorders, fifth Edition (DSM-5) [10] prohibits the diagnosis of catatonia when delirium is present. However, recent research has shown that these two syndromes can be comorbid, with estimates suggesting that 30–50% of older adults with catatonia in acute medical settings may also have coexisting delirium.

**Fig. 1:**
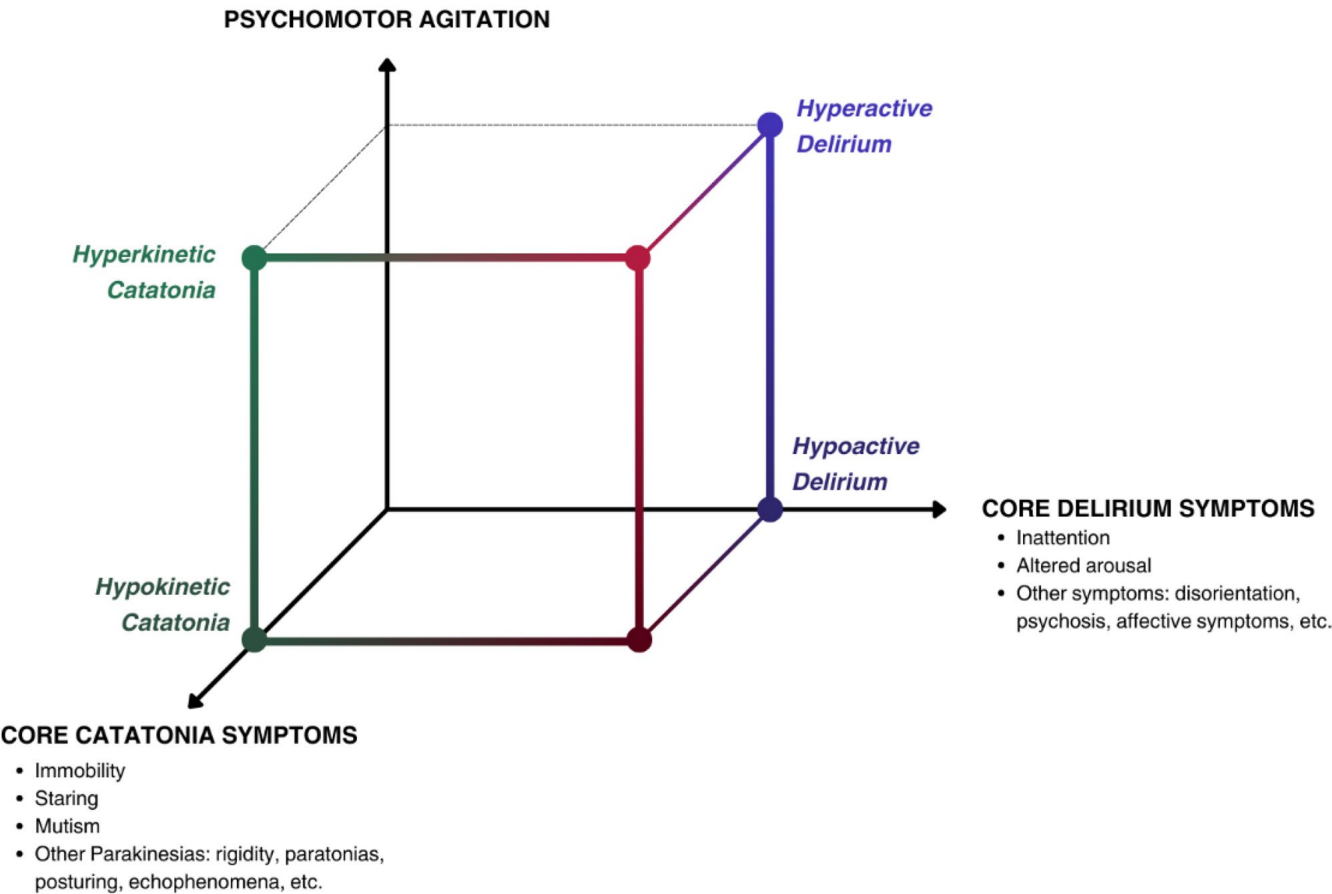
Conceptualizing acute brain dysfunction phenotypically in a cubic model: Overlap of delirium and catatonia psychomotor syndromes

 [11]. Comorbid delirium-catatonia syndrome (DeliCat) is a robust indicator of catatonia associated with an underlying physical health condition rather than a primary psychiatric one [12, 13]. In a sample of 136 critically ill patients, one-third had comorbid delirium and catatonia, compared to 3% only with catatonia alone [14]. It seems that the presence of both syndromes predicts worse outcomes [15]. Delirium and catatonia can even be seen in comatose patients, suggesting a multidimensional model for acute brain dysfunction [14]. The suggestion that catatonia and delirium are different yet overlapping manifestations of an underlying encephalopathy has been heavily suggested in the literature [14, 16–18]. Both syndromes represent phenotypes of acute brain dysfunction with similar psychomotor subtypes [18]. We represent this multidimensional concept in Fig. 1.

The diagnosis of catatonia or delirium is a clinical one, using DSM-5 or ICD-11 criteria. However, clinicians often rely on specific scales such as the Bush-Francis Catatonia Rating Scale (BFCRS) and the Confusion Assessment Method (CAM) for assessment of catatonia and delirium respectively [2, 8]. The BFRCS largely outperforms the DSM-5 criteria in terms of interrater agreement and pick-up rate [19]. This 23-item scale also provides the opportunity to evaluate treatment efficacy, especially following a lorazepam challenge test [2]. However, in patients with delirium, a higher cutoff (≥ 4) has been suggested to decrease overdiagnosis of catatonia [14]. For delirium, there is an agreement between the CAM and DSM-5 criteria, although the latter is more restrictive [20].

Research on catatonia is scarce globally but even more so in the Arab region. To our knowledge, only one study conducted in Kuwait has been published on the impact of ethnic differences on the prevalence of catatonia in an inpatient psychiatry population [21]. No study has been conducted on catatonia presentations and correlates in acutely physically ill patients on the consultation-liaison service in our region. In this paper, we describe confirmed cases of catatonia encountered on the Consultation-Liaison Psychiatry service (CLP) at a tertiary healthcare center in Lebanon. Additionally, we explore the correlates of catatonia with and without diagnosed delirium in physically ill patients. We conclude with a conceptual model of the DeliCat syndrome.

## Methods

After receiving approval from the American University of Beirut Institutional Review Board (BIO-2024-0176) which waived obtaining consent from subjects, we obtained access to the electronic health records of patients who fulfilled the following inclusion criteria: aged 18 or older; admitted to the hospital; seen by the Consultation-Liaison Psychiatry (CLP) team at least once; and “catatonia” or “catatonic” is mentioned in any note at least once. The charts used were from February 2018 (date of inception of CLP service) till May 27, 2024.

**Fig. 2:**
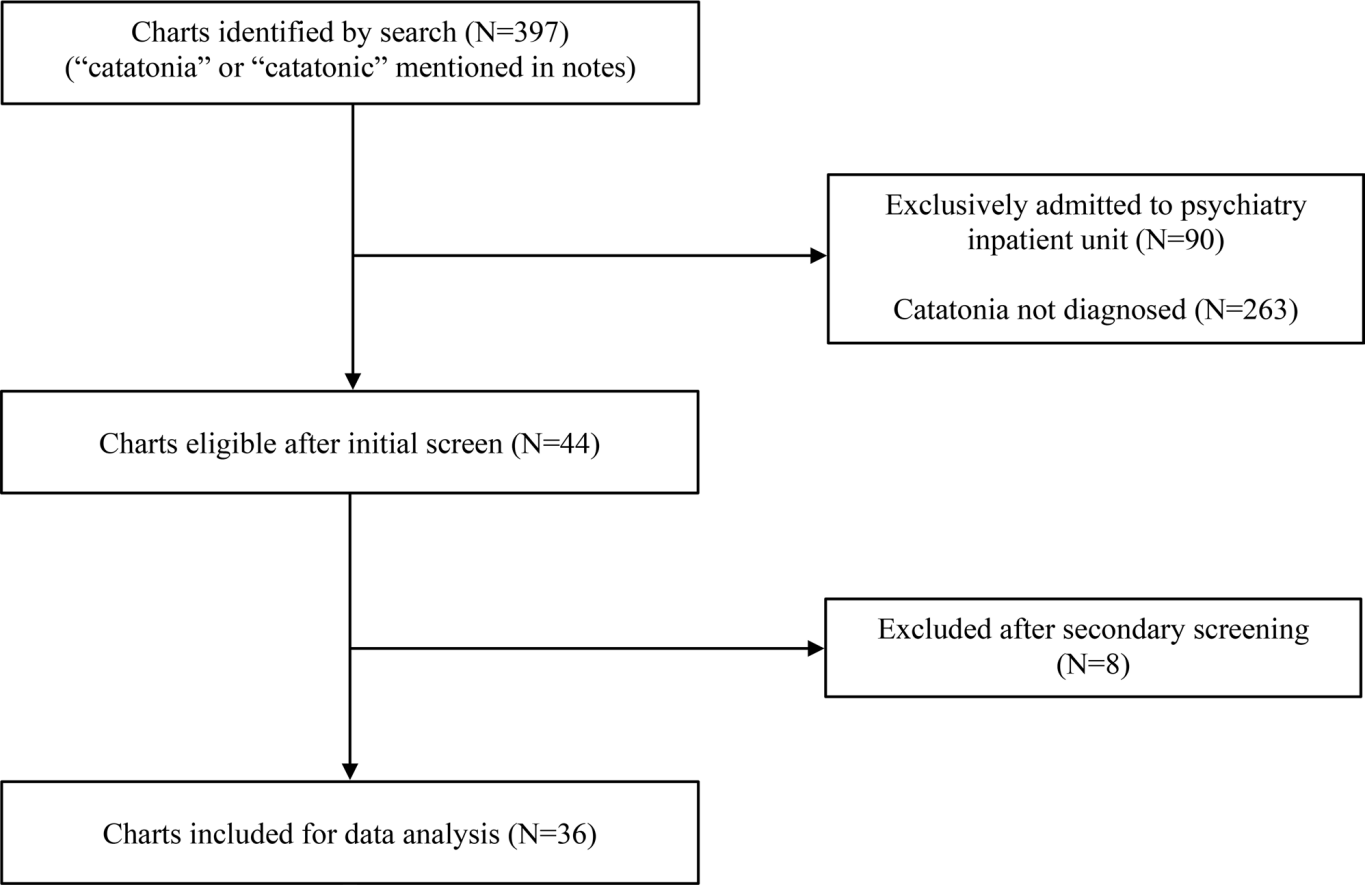
Flowchart depicting the screening process of patient charts

We received a total of 397 charts that were subsequently screened. We included charts indicating a confirmed diagnosis of catatonia due to a general medical condition by the CLP team using the DSM-5 criteria, which requires the presence of at least three out of twelve psychomotor features. Charts of patients who were exclusively admitted to the Psychiatry Inpatient Unit were excluded, as our unit only receives patients who are medically cleared. Figure 2 depicts the screening process. Patients with catatonia attributed solely to a psychiatric origin or chronic catatonia from an underlying psychiatric diagnosis (e.g., neurodevelopmental) were excluded. A secondary screening involved a more detailed chart review to exclude patients in whom catatonia was mentioned in the notes, but the diagnosis was not ascertained by the CLP team. After data collection was completed, one of the authors re-conducted data collection on 8 charts picked randomly, and their collected data were compared to the initially collected data. No inconsistencies were found between investigators.

Data was collected for the following variables: age, sex, medical history, history of traumatic brain injury, the reason for admission, active thrombotic problems (e.g., thrombotic stroke, deep vein thrombosis, myocardial infarction, pulmonary embolism), active brain problems (e.g., encephalitis, stroke), length of stay, the unit patient was admitted to, home medications, hospital medications, discharge medications, Bush-Francis Rating Scale score, specific catatonia signs and symptoms, presence of a delirium diagnosis according to the DSM-5 criteria, death during admission, treatments received for catatonia, imaging, laboratory, and electroencephalographic (EEG) results when performed. Data closest to the time of the first Psychiatry consult for catatonia were used. The category of patients with a dual diagnosis of delirium and catatonia is referred to as “DeliCat syndrome” throughout the manuscript.

The Statistical Package for the Social Sciences (SPSS) version 25.0 [22] was used for data cleaning, management, and analysis. Descriptive statistics are expressed as mean and Standard Deviation (± SD) for continuous variables, and frequencies and percentages for categorical variables. Association between catatonia, with and without comorbid delirium, and other categorical variables was carried out using Fisher’s exact test, whereas the Mann-Whitney U Test was used for the association with continuous variables. P-value < 0.05 was used to indicate statistical significance.

## Results

Our sample consisted of 36 individuals with catatonia diagnosis according to the DSM-5 criteria, out of which 18 (50%) had comorbid delirium (DeliCat syndrome). The characteristics of the population are presented in Table 1, stratified by catatonia with and without comorbid delirium. The mean age of our participants was 55.5 ± 19.1 years. They were almost equally distributed between sexes, 52% were males. Additionally, 52% of participants were smokers. Around one-third of patients had a history of non-psychiatric brain disease (27%), and around a third had a history of psychiatric diagnoses (36%). Half (50%) of the patients had no pre-existing brain disease (psychiatric and non-psychiatric). Around a fifth (22%) of the participants had a positive history of thrombotic disorders (e.g., history of ischemic stroke, myocardial infarction, pulmonary embolism).

When the two groups were compared, older age was significantly associated with DeliCat syndrome compared to catatonia alone (*p* = 0.006). In terms of past medical history, only history of thrombotic disorders was significantly associated with DeliCat syndrome (38%) compared to catatonia alone (5%) (*p* = 0.04). Patients with DeliCat syndrome had only numerically higher rates of chronic kidney disease, cardiovascular risk factors, and cancer compared to those with catatonia alone.

**Table 1:** Comparison of baseline characteristics between patients with catatonia only versus delicat syndrome

	Total *N* = 36	Catatonia alone *N* = 18	DeliCat *N* = 18	*p*-value
**Age**, mean ± SD	55.5 ± 19.1	46.6 ± 19.1	64.3 ± 14.8	**0.006**
**Sex**, Male	19 (52%)	10 (55%)	9 (50%)	1.00
**Smoker**	19 (52%)	10 (55%)	9 (50%)	1.00
**Cancer**	11 (30%)	4 (22%)	7 (38%)	0.47
**CKD**	9 (25%)	2 (11%)	7 (38%)	0.12
**Cardiovascular risk factors**	19 (52%)	8 (44%)	11 (61%)	0.50
**Non-psychiatric CNS disorders**	10 (27%)	5 (27%)	5 (27%)	1.00
**History of thrombotic disorders**	8 (22%)	1 (5%)	7 (38%)	**0.04**
**History of TBI**	3 (8%)	1 (5%)	2 (11%)	1.00
**Previous psychiatric diagnoses**	13 (36%)	6 (33%)	7 (38%)	1.00
**Positive history of catatonia**	2 (5%)	2 (11%)	0 (0%)	0.49

CKD: Chronic Kidney Disease; CNS: central nervous system; TBI: Traumatic Brain Injury; SD: Standard Deviation

The comparison of factors related to the hospital stay is presented in Table 3. There were no statistical differences concerning factors related to the hospital stay (e.g., length of stay, admission to critical care unit) between patients with catatonia only versus DeliCat syndrome. However, two findings are worth mentioning due to their clinical significance. The first is the abrupt discontinuation of psychotropic or antiseizure medications which was 4 times higher in the catatonia-only group compared to the DeliCat group. The second is mortality within 6 months, which is seen in 38% of patients with DeliCat syndrome compared to 16% of cases with catatonia only, highlighting the severity of the dual diagnosis (Table 2).

**Table 2:** Comparison of factors related to the hospital stay between patients with catatonia only versus DeliCat syndrome

	Total *N* = 36	Catatonia alone *N* = 18	DeliCat *N* = 18	*p*-value
Acute thrombotic disorders	7 (19%)	4 (22%)	3 (16%)	1.00
Acute CNS disorders	13 (36%)	7 (38%)	6 (33%)	1.00
Other acute medical problems	30 (83%)	13 (72%)	17 (94%)	0.17
Reason for admission: CNS-related	13 (36%)	8 (44%)	5 (27%)	0.49
Reason for admission: Thrombotic disorders	5 (13%)	4 (22%)	1 (5%)	0.33
Length of stay (days), mean ± SD	25.1 ± 20.4	29.1 ± 25.9	21.2 ± 12.3	0.79
Admission to critical care unit	19 (52%)	10 (55%)	9 (50%)	1.00
Admission to PIU	5 (13%)	4 (22%)	1 (5%)	0.33
Abrupt discontinuation of psychotropic or antiepileptic medications	10 (27%)	8 (44%)	2 (11%)	0.06
Mortality within 6 months	10 (27%)	3 (16%)	7 (38%)	0.26

CNS: central nervous system; PIU: Psychiatry Inpatient Unit; SD: Standard Deviation

The catatonic signs for both groups are presented in Table 3. There is no difference in the psychomotor subtypes between the groups. While all patients except one in the catatonia-only group were treated with benzodiazepines, only 55% of patients with DeliCat were given benzodiazepines. Moreover, the DeliCat group received significantly lower lorazepam equivalence doses throughout the treatment duration (55% vs. 94%, p-value = 0.007). The average for each initial, maximum, and discharge Lorazepam equivalence showed significant differences between the two groups (*p* = 0.004, *p* = 0.002, and *p* = 0.009, respectively). None of the patients with DeliCat underwent Electroconvulsive Therapy (ECT).

**Table 3:** Comparison of catatonic signs and symptoms, and treatment parameters between patients with catatonia only versus DeliCat syndrome

	Total *N* = 36	Catatonia alone *N* = 18	DeliCat *N* = 18	*p*-value
Excitement present	9 (25%)	5 (27%)	4 (22%)	1.00
Paliphenomena present	18 (50%)	9 (50%)	9 (50%)	1.00
Paratonia present	15 (41%)	9 (50%)	6 (33%)	0.50
Immobility present	24 (66%)	14 (77%)	10 (55%)	0.28
Rigidity present	22 (61%)	14 (77%)	8 (44%)	0.86
Ambitendency present	9 (25%)	4 (22%)	5 (27%)	1.00
Automatic obedience present	13 (36%)	6 (33%)	7 (38%)	1.00
Mutism present	27 (75%)	13 (72%)	14 (77%)	1.00
Echophenomena	9 (25%)	4 (22%)	5 (27%)	1.00
Grasp Reflex present	10 (27%)	4 (22%)	6 (33%)	0.71
Busch Francis Scale on Diagnosis, mean ± SD	16.88 ± 6.59	18.53 ± 7.18	15.41 ± 5.85	0.23
Mixed Psychomotor Presentation	11 (30%)	5 (27%)	6 (33%)	1.00
Received Lorazepam	27 (75%)	17 (94%)	10 (55%)	**0.007**
Lorazepam equivalence, mg Initial, mean ± SD	1.53 ± 1.5	2.18 ± 1.52	0.87 ± 1.11	**0.004**
Lorazepam equivalence, mg Maximum, mean ± SD	3.84 ± 5.1	6.0 ± 6.2	1.7 ± 2.5	**0.002**
Lorazepam equivalence, mg Discharge, mean ± SD	1.36 ± 2.1	1.9 ± 1.9	0.8 ± 2.1	**0.009**
Time till resolution after starting BZD, mean ± SD	7.1 ± 6.0	8.4 ± 6.2	5.0 ± 5.7	0.16
Required ECT	3 (8%)	3 (16%)	0 (0%)	0.22

BZD: benzodiazepine; ECT: electroconvulsive therapy; mg: milligrams; SD: standard deviation

Patients with DeliCat syndrome were significantly less likely to undergo a lumbar puncture (LP) (16% vs. 72%, *p* = 0.002). No differences were observed in the percentage of abnormal LP or EEG between the two groups. It is worth mentioning that three DeliCat patients (27%) had epileptiform discharges on EEG compared to zero catatonia-only patients. No differences were observed concerning the presence of slowing of the background on EEG. Abnormal imaging was common in both groups (70%). Three patients in each group had new vascular lesions on imaging (Table 4).

**Table 4:** Differences in procedures and imaging between catatonia-only and DeliCat patients

	Total *N* = 36	Catatonia alone *N* = 18	DeliCat *N* = 18	*p*-value
LP done	16 (44%)	13 (72%)	3 (16%)	**0.002**
Abnormal LP	6 (37%)	5 (38%)	1 (33%)	1.00
EEG Done	26 (72%)	15 (83%)	11 (61%)	0.26
Generalized Slowing on EEG	22 (84%)	13 (86%)	9 (81%)	1.00
Epileptiform on EEG	3 (11%)	0 (0%)	3 (27%)	0.06
New Vascular lesions	6 (17%)	3 (17%)	3 (17%)	1.00
Abnormal imaging	24 (70%)	12 (70%)	12 (70%)	1.00

EEG: Electroencephalogram; LP: lumbar puncture

Concerning laboratory values, no statistical difference was observed between the two groups except for creatinine and blood urea nitrogen (BUN). Both were significantly higher in the DeliCat group as compared to catatonia-only (1.6 ± 1.4 vs. 0.8 ± 0.6, *p* = 0.02 and 41.0 ± 35.7 vs. 22.8 ± 24.3, *p* = 0.02, respectively). However, no statistical significance was observed in the BUN to Creatinine ratio. Results are presented in Table 5.

**Table 5:** Differences in laboratory values between catatonia only and DeliCat patients

	Total *N* = 36	Catatonia alone *N* = 18	DeliCat *N* = 18	*p*-value
**CPK**, mean ± SD	182.1 ± 195.6	219.0 ± 212.2	141.0 ± 178.4	0.59
**WBC**, mean ± SD	10.0 ± 5.7	9.6 ± 3.8	10.4 ± 7.2	0.91
**Hct**, mean ± SD	33.9 ± 6.3	35.5 ± 5.1	32.2 ± 7.0	0.10
**MCV**, mean ± SD	88.1 ± 8.9	87.1 ± 9.8	89.0 ± 8.1	0.85
**Neutrophils Ratio**, mean ± SD	0.7 ± 0.1	0.7 ± 0.1	0.76 ± 0.12	0.08
**Lymphocyte Ratio**, mean ± SD	0.2 ± 0.1	0.2 ± 0.08	0.2 ± 0.18	0.16
**Neutrophils to Lymphocyte Ratio**, mean ± SD	7.9 ± 10.8	6.5 ± 10.7	9.4 ± 11.0	0.14
**Glucose**, mean ± SD	144.7 ± 52.8	150.1 ± 61.8	139.4 ± 43.1	0.91
**BUN**, mean ± SD	31.9 ± 31.5	22.8 ± 24.3	41.0 ± 35.7	**0.02**
**Creatinine**, mean ± SD	1.2 ± 1.1	0.8 ± 0.6	1.6 ± 1.4	**0.02**
**BUN-to-Cr Ratio**, mean ± SD	27.0 ± 14.9	25.2 ± 165	28.7 ± 13.5	0.27
**Sodium**, mean ± SD	137.7 ± 6.3	136.6 ± 4.8	138.9 ± 7.5	0.14
**Potassium**, mean ± SD	4.0 ± 0.6	4.1 ± 0.6	4.0 ± 0.5	0.53
**Chloride**, mean ± SD	101.4 ± 6.2	101.4 ± 4.7	101.39 ± 7.5	0.89
**Bicarbonate**, mean ± SD	23.1 ± 3.2	22.6 ± 3.7	23.6 ± 2.8	0.39
**Magnesium**, mean ± SD	1.9 ± 0.4	2.0 ± 0.3	1.9 ± 0.4	0.29
**Calcium**, mean ± SD	8.9 ± 0.7	9.0 ± 0.7	8.8 ± 0.8	0.33
**Phosphorus**, mean ± SD	3.4 ± 1.2	3.3 ± 1.0	3.6 ± 1.3	0.65
**ALT**, mean ± SD	50.6 ± 67.5	44.7 ± 44.5	56.2 ± 84.8	0.63
**TSH**, mean ± SD	3.2 ± 5.1	2.7 ± 3.0	3.9 ± 7.0	0.94
**Procalcitonin**, mean ± SD	0.5 ± 0.6	0.3 ± 0.4	0.6 ± 0.6	0.13
**CRP**, mean ± SD	61.7 ± 93.6	60.5 ± 97.0	62.7 ± 93.5	0.77

CPK: Creatine Phosphokinase; WBC: White Blood Cells; Hct: Hematocrit; MCV: Mean Corpuscular Volume; BUN: Blood Urea Nitrogen; ALT: Alanine Aminotransferase; TSH: Thyroid-Stimulating Hormone; and CRP: C-Reactive Protein

## Discussion

Literature on catatonia on consultation-liaison services is scarce, which limits the comparison of our data with existing literature. Studies on catatonia due to general medical illness report that most cases (68–77%) happen in the context of neurological disorders [17, 23]. This contrasts with our findings as only around 36% of our patients were found to have active neurological problems, despite being extensively worked up. While a direct encephalopathic insult (e.g., encephalitis, drugs) is a robust precipitant of catatonia, our sample suggests that indirect encephalopathic insults (e.g., through systemic inflammation) may be just as relevant.

Most of the EEGs done for our sample were abnormal, with 22 out of the 26 EEGs (84%) showing a generalized slowing of background activity, and a tenth having epileptiform discharges. Such abnormal findings were reported in previous cases of catatonia due to general medical illness, reaching 92% in one study [23]. Another study found the rate of generalized slowing to be 80%; the authors proposed that this lends validity to a delirium-catatonia spectrum [17]. Abnormal brain imaging was observed in 70% of our cases, a percentage slightly higher than in previous studies [13, 23].

The remaining blood investigations did not yield any promising findings to discriminate catatonia from DeliCat syndrome in our sample. While an elevated BUN-to-creatinine ratio has been reported in patients with delirium compared to non-delirious patients [24], specifically in patients with hypoactive delirium [25], data overall is scarce. In our sample, both BUN and creatinine were significantly higher in the DeliCat group, possibly attributed to high CKD comorbidity in this group at baseline (38%), compared to the catatonia-only group (11%). However, no statistical significance was observed in the BUN-to-Creatinine ratio between the two groups. Pragmatically, there seems to be no readily available biomarker that indicates a diagnosis of catatonia, delirium, or both. Diagnoses rely on the clinical assessment of phenotypes, although this is limited by the nature of the delirium and catatonia syndromes. Moreover, while helpful at times, diagnostic systems and tools are imperfect [2, 8, 15, 18].

The most observed catatonic signs in our sample were mutism (75%) and immobility (66%). This is similar to previously described findings in patients admitted to general medical floors, where those two signs were consistently reported in more than two-thirds of patients [13, 26, 27]. This is also consistent with the newest research diagnostic criteria (RDCC) which suggests akinetic symptoms (mutism, immobility, and staring) as the core symptoms of catatonia in the physically ill [28]. Other symptoms like echophenomena and stereotypies are considered less common and less specific [29]. The focus of the RDCC on akinetic symptoms promises increased diagnostic accuracy of catatonia, regardless of the underlying cause, with little risk of confounding from nonspecific behavioral signs. This is especially true when delirium is comorbid because tools such as BFCRS can overdiagnose cases. In fact, the RDCC excludes excitement as a feature of the syndrome and attributes it to comorbid delirium. Even in cases when excitement is associated with unusual behavioral signs (e.g., stereotypy), the RDCC proposes this may represent a distinct syndrome [28].

Nevertheless, the RDCC acknowledges the overlapping phenomenology of delirium and catatonia. Our findings support the proposition that both delirium and catatonia belong to the same spectrum of neuropsychiatric dysfunction. We propose that the manifestation of either or both depends on two factors: brain vulnerability and adaptive/maladaptive sickness responses, as illustrated in Table 6.

**Table 6:** Examples of proposed adaptive sickness responses in a healthy versus vulnerable brain

Adaptive Responses	Healthy Brain	Vulnerable Brain
Sickness Behavior Syndrome	Fatigue, loss of appetite, social withdrawal, sleep disturbances, malaise, and difficulty concentrating	Lethargy, psychomotor slowing, disturbed circadian rhythm, depressed mood, and inattention
Fight-or-Flight Response	Muscle tension/rigidity, heightened vigilance, fear, anxiety, anger, and impulsivity	Excitement, impulsivity, combativeness, disorganized behavior, and paranoia
Freeze Response	Muscle tension/rigidity, heightened vigilance, fear, anxiety, anger, immobility, feeling trapped, and reduced sense of control/agency	Mutism, immobility, staring, negativism, paratonias, and waxy flexibility

### Brain vulnerability

Half of our catatonia cases had comorbid delirium. This was lower than what was reported in the “DeCat prospective cohort study” [14] in which 90% of their catatonic patients had delirium. The higher rate can be attributed to their older population and the inclusion of solely critically ill patients in their cohort. Moreover, the predominance of the hypokinetic catatonia presentation in our patients (e.g., stupor, mutism) could have prevented a comprehensive clinical assessment of delirium, which can potentially underestimate the comorbidity.

Several delirium risk factors are established in the literature, including older age, dementia, depression, frailty, and high comorbidity burden [8]. While our study lacks statistical power to evaluate the impact of these vulnerability factors on developing delirium, we observed a trend for higher rates of comorbidities and 6-month mortality in our DeliCat population. In addition, older age was significantly correlated with developing DeliCat compared to catatonia alone in our sample [30]. Moreover, the DeliCat group had a significantly increased history of thrombotic events compared to patients with catatonia alone, which postulates a possible role of poor vascular health and blood-brain barrier disruption in predisposing individuals to delirium [31, 32]. As for catatonia, the presence of an existing neuropsychiatric disorder (e.g., bipolar disorder, neurodevelopmental disorder, structural insults) is shown to be a predisposing factor [8]. This is consistent with our sample, where 50% have a history of a brain disorder (psychiatric or non-psychiatric).

It seems that the central nervous system can be vulnerable to developing either delirium, catatonia, or both, depending on the predisposing factors involved, which need further characterization in future research studies.

### Adaptive and maladaptive sickness responses

In a healthy brain, sickness induces normal evolutionary-adaptive responses which include the sickness behavior syndrome (SBS) [33] and the defense cascade (fight-or-flight-or-freeze responses) [34]. These responses are products of neuroinflammation [33] and limbic-hypothalamic-pituitary axis dysregulation [34], respectively.

Sickness behavior syndrome (SBS) shares common symptom clusters and neuroinflammatory underpinnings with delirium, including secretion of pro-inflammatory cytokines and microglial activation [8, 33, 35]. Clinically, a study reported that 96% of patients with delirium had symptoms of SBS. The authors proposed that delirium and SBS are part of the same behavioral continuum [36]. The proposition that delirium is a maladaptive manifestation of SBS is well established in the literature, and the development of one or the other depends on brain vulnerability [37–39], like older age or pre-existing dementia [36].

Sickness also induces the activation of sympathetic nervous systems leading to the fight-or-flight response [40–43]. In vulnerable individuals, these systems can lead to hyperactive states [33, 44] like so-called excited deliriums [45]. The distinction between hyperactive delirium and hyperactive catatonia is unclear both clinically and conceptually [5, 46] with syndromes like *delirium tremens* and malignant catatonia posing a diagnostic challenge [5, 6]. These overlapping excited states may in fact represent a hyperactive form of DeliCat. In general, hyperactive states can alternate episodically with hypokinetic catatonia or hypoactive delirium (so-called “mixed” subtypes) [28], although they can also happen alone [44, 47, 48].

The freeze response is another element of the normal defense cascade. It shares the same core akinetic features with catatonia [34], which led researchers to suggest that hypokinetic catatonia is an aberrant activation of the freeze response [49–51]. This is also consistent with the RDCC [28]. It is further supported by studies of patients’ subjective experience of catatonia which features intense fear, ambivalence, and inability to move [52–54]. Newer proposed social-defense behaviors (e.g., fawn, faint) [55] may be similarly involved in catatonia pathogenesis, but little literature has been published on the matter.

**Fig. 3:**
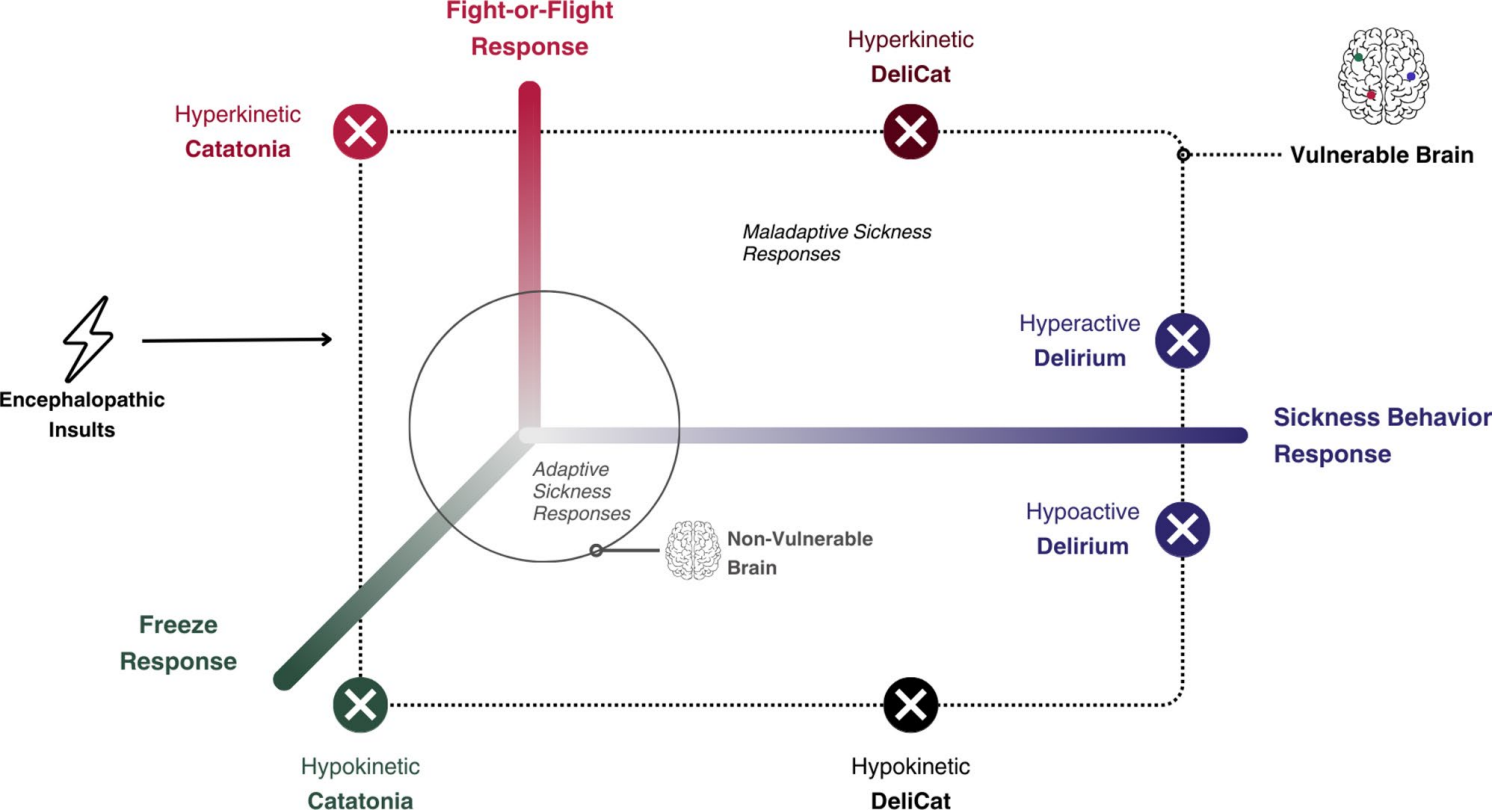
The Maladaptive Sickness Response (MSR) Model: When a vulnerable brain is faced with an encephalopathic insult, evolutionary-adaptive sickness responses malfunction, leading to delirium and catatonia syndromes

Therefore, we propose the Maladaptive Sickness Response (MSR) model which suggests that delirium and catatonia syndromes manifest when normal adaptive responses malfunction in a vulnerable brain (Fig. 3). This suggestion has been proposed by emerging literature on evolutionary psychiatry and brain-immune system interaction [33, 37, 44, 49, 56], but the overlap of the two syndromes has been overlooked [37–39]. Therefore, after an encephalopathic insult in vulnerable brains, core delirium symptoms may represent an aberrant manifestation of SBS, and core symptoms of hypokinetic catatonia, an aberrant manifestation of the freeze response. Hyperkinetic forms may represent an aberrant fight-or-flight response. The MSR model is a step towards developing a clinically pragmatic model grounded in evolutionary psychiatry.

### Limitations and future research

Our study is not without limitations: it is a retrospective chart review of electronic health records stemming from a single center, with a small sample size, leading to low statistical power. Hence, logistic regression could not be conducted. Moreover, diagnoses of both catatonia and delirium were done using the DSM-5 criteria with additional assessment tools such as the CAM or BFCRS not being readily available for all patients. Despite these limitations, our study is unique in terms of exploring an understudied topic. We recommend building on our observations to guide future research studies pertaining to catatonia in the physically ill.

First, we recommend conducting larger studies to better ascertain the vulnerability and precipitating factors of catatonia and delirium within the MSR model where catatonia and delirium overlap. The testing of multiple comparisons in a small sample increases the risks of false positives, which limits the generalizability of our study. Second, we recommend validating and ascertaining the diagnostic accuracy of existing tools to reliably differentiate between different syndromes. These tools can be guided by the RDCC and MSR model, basing the observations on adaptive versus maladaptive stress responses. Finally, we recommend using more advanced neuroimaging or inflammatory biomarkers assessments in future studies on the DeliCat versus delirium versus catatonia syndromes to enhance diagnostic accuracy and treatment management of these different phenotypic presentations.

## Conclusion

Catatonia in the physically ill is an understudied topic. Its comorbidity with delirium is an emerging concept that is increasingly being explored. We hypothesize that delirium, catatonia, and DeliCat occur on a continuum of a maladaptive stress response model, that needs further conceptualization, and validation using reliable and valid tools.

## Data Availability

The datasets used and/or analyzed during the current study are available from the corresponding author on reasonable request.
